# Odor Discrimination by Lipid Membranes

**DOI:** 10.3390/membranes13020151

**Published:** 2023-01-24

**Authors:** Troy W. Lowry, Aubrey E. Kusi-Appiah, Debra Ann Fadool, Steven Lenhert

**Affiliations:** Department of Biological Science, Florida State University, Tallahassee, FL 32306, USA

**Keywords:** odorant, enantioselectivity, lipid, droplet, microarray, biosensor, nose, lithography, nanointaglio

## Abstract

Odor detection and discrimination in mammals is known to be initiated by membrane-bound G-protein-coupled receptors (GPCRs). The role that the lipid membrane may play in odor discrimination, however, is less well understood. Here, we used model membrane systems to test the hypothesis that phospholipid bilayer membranes may be capable of odor discrimination. The effect of S-carvone, R-carvone, and racemic lilial on the model membrane systems was investigated. The odorants were found to affect the fluidity of supported lipid bilayers as measured by fluorescence recovery after photobleaching (FRAP). The effect of odorants on surface-supported lipid multilayer microarrays of different dimensions was also investigated. The lipid multilayer micro- and nanostructure was highly sensitive to exposure to these odorants. Fluorescently-labeled lipid multilayer droplets of 5-micron diameter were more responsive to these odorants than ethanol controls. Arrays of lipid multilayer diffraction gratings distinguished S-carvone from R-carvone in an artificial nose assay. Our results suggest that lipid bilayer membranes may play a role in odorant discrimination and molecular recognition in general.

## 1. Introduction

Odor detection in mammals is initiated in cellular membranes that contain well-studied G-protein-coupled receptors (GPCRs) [1,2,3,4,5]. Upon exposure to odorant molecules, these membrane-bound proteins trigger several signaling events, many of which also occur in the same membrane, which eventually lead to pattern recognition of the odor by the brain. Since the discovery that olfactory receptors are GPCRs, significant efforts have been made to identify odorant binding sites through the development of better expression systems and functional assays, with emphasis on olfactory receptor binding activity and sensitivity [6,7,8,9,10,11,12]. Unlike some other GPCRs [13], the olfactory receptor binding site has not been fully identified [14,15]. While there has been a lot of work done to model the odor binding site, there is uncertainty about how the odors reach and activate them, as well as how they might modulate downstream signaling events [16,17,18,19,20]. This leaves a gap in our molecular understanding of odorant interactions in the olfactory epithelium. A more complete understanding of molecular recognition of odorants is important for understanding health-related issues such as anosmia [21] and olfactory sense perception [20,22,23]. Furthermore, a better understanding of the role of the membrane in olfactory receptor activation will have broader applications for developing drug targets in the pharmaceutical industry of which GPCRs make up a large component [24,25]. The goal of this study was to test the idea that the cell membrane lipid composition may play a role in the odor discrimination process through selective interactions with odorants. This way of thinking about lipid bilayers as a supramolecular receptor for odor recognition is new, as selective odorant binding to GPCRs is generally hypothesized to be the main mechanism.

In a number of cases, membrane composition and structure are thought to regulate membrane protein activity [26,27]. Mechanosensitive ion channels such as MscL are known to open in response to mechanical perturbation of the membrane [28,29]. The activity of several GPCRs has been found to be regulated by lipid composition, for instance the cholesterol content of the lipid membrane [30,31,32]. Combinations of lipids have been proposed to play more than just a supporting role for proteins in biological systems [33,34,35]. For instance, in a model system it was shown that aggregates of lipid-like molecules can demonstrate molecular recognition as a kind of supramolecular aptamer [35]. There is evidence that lipids may play a role in the detection of odorants [36,37,38]. It has been shown that lipid monolayers from bovine epithelium have a change in surface tension that is linearly correlated to odorant threshold concentration [36]. Furthermore, it has been shown that olfaction can be temperature-sensitive [39,40]. Further experiments showed a strong correlation with the odorant’s effect on membrane fluidity [39].

Chemically, odorants tend to be relatively small hydrophobic compounds [41]. This is typically explained as being due to the need for volatility as well as crossing the olfactory mucosa. It is reasonable to hypothesize that these hydrophobic molecules may also accumulate in lipid bilayers. Enantioselectivity is a striking property of the olfactory system, with enantiomeric odorants having distinctly different odors [42]. Herein, we test the hypothesis that the lipid membranes not only play an elementary structural role for odor receptors but may also participate in odor discrimination.

Model membrane systems such as vesicles and supported lipid bilayers are well established for studying the behavior of lipid bilayers in vitro [43,44,45,46]. Supported lipid bilayers can demonstrate fluidity, although often interactions with the underlying substrate result in fluidity differences compared with bilayers in vesicles [47,48]. Being attached to a surface makes surface-supported model membrane systems better suited for optical microscopy, for instance upon the addition of reagents. Surface-supported lipid multilayers or droplet arrays are a newer model membrane system that combine the advantages of a surface-based system with some of the three-dimensional properties of vesicles [49,50,51,52,53,54,55]. These systems have been used for miniaturized drug screening in cell culture [49,50], for biosensing elements [52,54,56], and as a model system to understand protein–lipid interactions [52]. Lipid droplet arrays have been utilized to monitor membrane binding events of streptavidin [52,55], the ER membrane binding protein Sar1 [55], as well as interactions with volatile compounds [54]. Microscopic structural changes can be monitored using fluorescently labeled lipid additives [52,55,57]. Nanoscopic structural changes can also be monitored by observing optical diffraction from diffraction gratings formed out of the lipid droplets [52].

In the case of lipid multilayer gratings, nanoscale heights and optically efficient periodicity along the wavelength of light can be patterned onto a surface, and diffraction intensity can be monitored from the lipid grating as a function of time for measuring and modeling of analyte activity [52,54,55]. Lipid multilayer gratings are a cost-efficient and time-conserving method that can be used with any camera and angled white light source in order to study the membrane binding and remodeling activity of proteins. The benefit of using these techniques is that they are massively parallel and have considerable material integration capability [49,58].

Lipid micro- and nano-structured droplets have formed on surfaces by dip-pen nanolithography [52,53,59,60,61], polymer-pen lithography [62], evaporative edge lithography [63], and nanointaglio printing [58,64,65]. Nanointaglio printing is the method used here, which involves printing lipid inks from micro- or nanostructured stamps, with the ink being transferred from the recesses of the stamps [58,64,65]. This process is capable of making droplet arrays as well as diffraction gratings from lipid inks of various formulations. In several cases, the height or thickness of the lipid droplets on the surface have been found to be a crucial parameter that relates to their sensitivity to analytes. Nanointaglio is capable of producing structures of varying heights by repeated printing, and as ink is depleted from the stamp the droplets produced have lower heights [58,64,65].

## 2. Materials and Methods

*Lipid multilayer substrate preparation*: Lipid multilayers of 1,2-dioleoyl-sn-glycero-3-phosphocholine (DOPC) doped with one mol percent rhodamine-1,2-dipalmitoyl-sn-glycero-3-phosphoethanolamine-N-(lissamine rhodamine B sulfonyl) (ammonium salt) (rhodamine-PE) were arrayed onto a clean glass surface using a polymeric stamp of either dots or gratings. As confirmed previously by atomic force microscopy, the dot lipid multilayers were around 250 nm in height and 5 µm in diameter, while the lipid gratings had 700 nm periodicity and sub-100 nm heights. They were immersed in deionized water, and then odorants were introduced in solution by reverse pipetting. A concentration of 1 µM carvone enantiomers (R—product number: 124,931 and S—product number: 435,739, Sigma Aldrich, St. Louis, MO, USA) caused disruption of pattern fidelity and dissolution compared with the application of ethanol at the same concentration on a different sample. Chemical structures were rendered using ChemDraw Pro v.8 for Windows and Powerpoint.

*Fluorescence recovery after photobleaching (FRAP)*: The purpose of FRAP on surface-supported lipid bilayers is to indicate a change in the recovery of the membrane (a measure of fluidity of the membrane) after exposure to the odorant. Due to carvone enantiomer’s low solubility in water, solutions were administered in a distilled water solution of 0.5% DMSO. DOPC with one mol percent Rhodamine-PE supported lipid bilayers were prepared using a vesicle fusion method by washing sonicated vesicles in deionized water (pH 7) with 1 M Ca^2+^ ions on an oxygen-plasma-coated glass slide (medium power, 2 min). Applying vesicles onto the surface with Ca^2+^ ions in solution and then washing caused the rupturing of vesicles onto the surface whereby the wash steps removed the excess Ca^2+^.

*Nanointaglio printing*: Lipid multilayer dots and gratings were fabricated by nanointaglio [58]. Dehydrated lipid formulations were transferred onto a polydimethylsiloxane (PDMS) grating stamp (700 nm pitch, 350 nm height) or PDMS hole stamp (5 µm diameter, ~1.8 µm depth) from an inked PDMS palette. Excess lipid ink was removed by sacrificial printing (proofing) [58,64]. The PDMS stamp was then stamped onto the substrate of choice.

*Lipid pattern storage*: After nanointaglio fabrication, lipid gratings and dots were stored in a nitrogen glovebox (Mbraun, Inc., Model Labstar (1200/780) Stratham, NH, USA) for 24 h to remove all water or to dehydrate them. The nitrogen environment stabilized the lyotropic lipid multilayer nanostructures by dehydration prior to immersion in water [52]. After immersion, the lipid multilayer gratings remained stable in buffer solution and remained physisorbed to the substrate.

*Lipid multilayer gratings vapor chamber*: Lipid multilayer gratings were arrayed onto 100 mm diameter polystyrene petri dishes. The gratings were stabilized in a set position using double-sided tape, then characterized using a diffraction light setup for a baseline control before odorant addition. We deposited 200 µL of carvone enantiomer (Sigma Aldrich) on a chem wipe into the corner of the chamber, and then the chamber was immediately sealed from the outside with Parafilm. Once sealed, diffraction images were taken every five seconds. Choosing time points at 20 and 40 s after odorant application enabled measurement of lipid multilayer responses to the odorants, which then allowed us to generate a point in principal components analysis (PCA). One hundred-pixel regions of interest (ROI)s were chosen from each of the six lipid grating formulations and used for the analysis. Three different samples were used for each carvone enantiomer. PCA was executed on the diffraction gratings as described previously [54] using Origin Pro.

## 3. Results and Discussion

**Figure 1:**
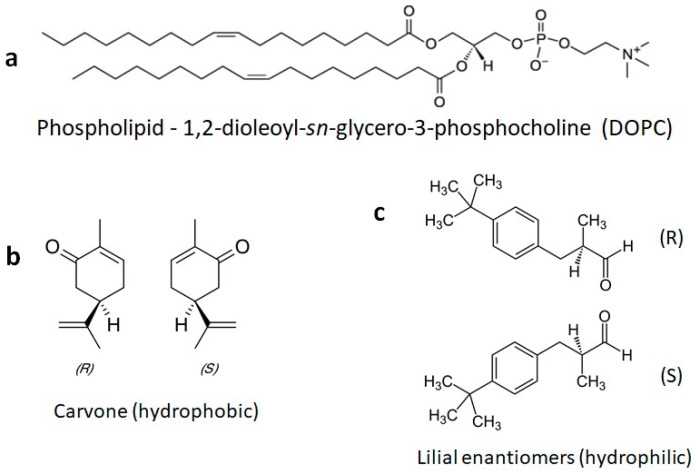
Chemical structures of a phospholipid and the odorants used here. (**a**) The phospholipid DOPC is commonly used to form fluid model membranes in water at room temperature. (**b**) The odorants S-carvone and R-carvone. (**c**) The odorant lilial, which was used here as a racemic mixture.

**Figure 2:**
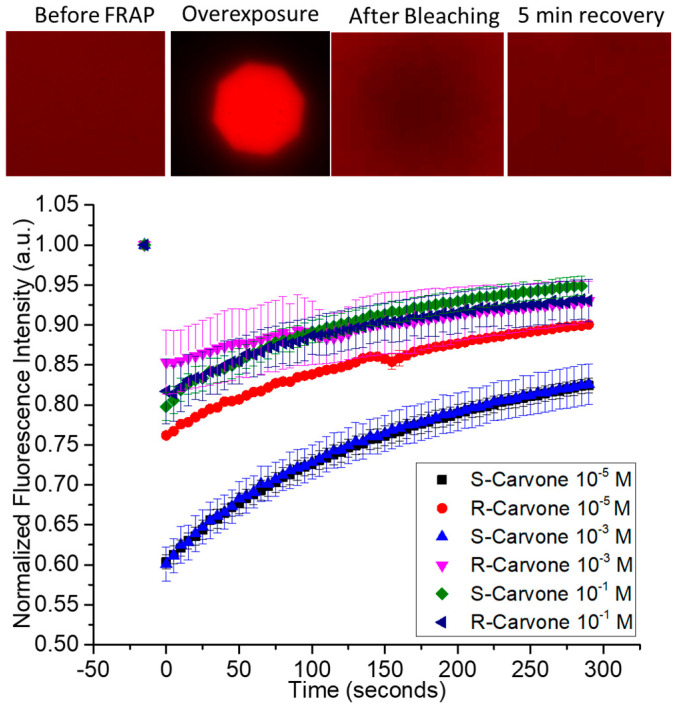
Fluorescence recovery after photobleaching of supported lipid bilayers upon exposure to enantiomeric odorants S-carvone and R-carvone. Top—examples of data from the FRAP experiment. A fluorescently labeled supported lipid bilayer was exposed to high-intensity light through an aperture to bleach a circular area within the spot. The fluorescence intensity of the bleached spot was measured as lipids diffused into it from the unbleached region. Represented are FRAP data from the two enantiomers, measured at 3 different concentrations.

The chemical structures of the lipids and odorants used here are shown in Figure 1. Phospholipids such as DOPC spontaneously form lipid bilayers in water. The central carbon of the glycerol component of the molecule contains a chiral carbon. The odorants carvone and lilial are both known to present different odors depending on their chirality. The effect of the introduction of an odorant on the fluidity of supported lipid bilayers was measured by fluorescence recovery after photobleaching (FRAP—Figure 2). Supported lipid bilayers were formed onto an oxygen-plasma-treated glass surface. After washing, a final solution with the carvone odorant in 0.5% DMSO was added to the solution. Initial images were captured and fluorescence intensity measured using ImageJ ROI analysis in the region of interest surrounding the bleached pinhole region. This intensity was compared to the initial fluorescence intensity before bleaching. Error bars represent the standard error from the average ROI intensity of each of the samples for each concentration.

Based on qualitative observation, the data suggest the concentration of carvone enantiomers may impact the fluidity of lipid bilayers differently, depending upon the enantiomer used. Specifically, there is a larger difference in the bleaching intensity for S-carvone 10^−5^ M and S-carvone 10^−3^ M when compared with the R-enantiomers at those concentrations. Since the diffusion of unbleached molecules continues during bleaching, this suggests that S-carvone decreases the fluidity of the bilayer compared with R-carvone at these concentrations. Surprisingly, this effect does not appear at the higher concentration (10^−1^ M). A possible explanation is that the lipid bilayer chirality interacts more with the S-enantiomer than the R-enantiomer to cause a decrease in the membrane exchange (lower fluorescence intensity) of the region outside membrane exposure, thus indicating differences in membrane fluidity at specific concentrations. Supplementary Figure S1 shows the FRAP of the hydrophilic odorant lilial in comparison with the FRAP of surface-supported DOPC bilayers without the presence of lilial. A typical recovery curve for the fluid phospholipid DOPC at room temperature conditions is presented in this figure and supports the interesting FRAP characteristic of the initial lower intensity of the overexposed bleached area and lack of complete fluorescent recovery, unlike the control.

**Figure 3:**
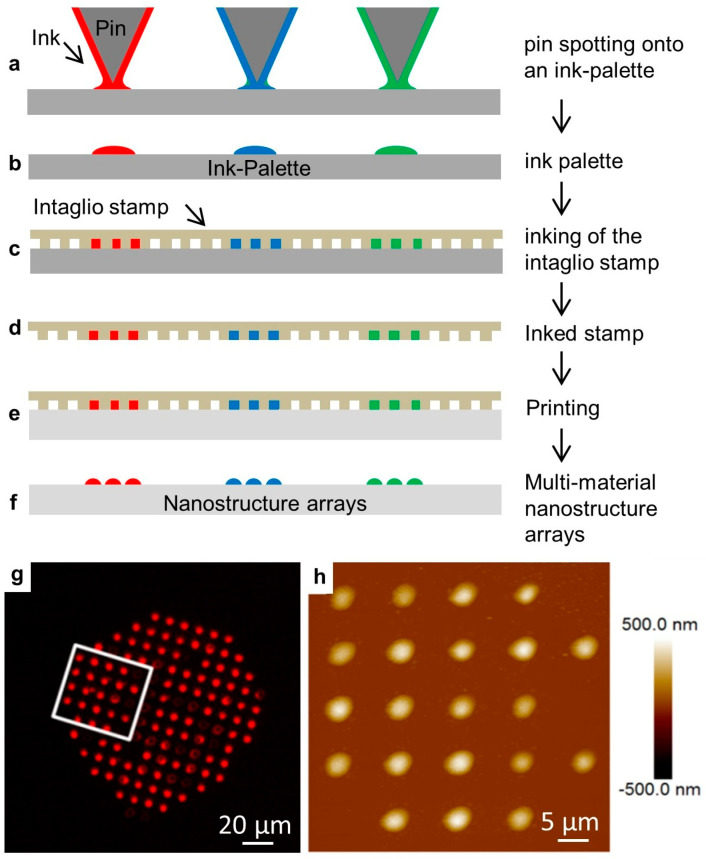
The nanointaglio printing process. (**a**–**f**) Schematic illustration of the nanointaglio process. (**a**,**b**) An ink palette is prepared by spotting inks onto surface. (**c**,**d**) A microstructured polymer stamp is inked by placing the stamp in contact with the palette. (**e**) The inked stamp is used to print from the recesses of the stamp. Initial prints are discarded as excess ink is removed from the surface of the stamp until intaglio printing occurs. (**f**) The printed droplet arrays. (**g**) Fluorescence micrograph of a printed droplet array. (**h**) Atomic force micrograph of the area outlined in (**g**).

We then tested the effect of odorant solutions on lipid multilayer droplet arrays immersed in water. Arrays were fabricated by nanointaglio as shown with examples in Figure 3. In this process, lipid inks are placed onto a palette using a pipette or pin-spotting tool. A microstructured PDMS stamp is then inked by placing the stamp onto the palette. Excess ink is removed by sacrificial printing, and then droplets are printed onto the surface. The process is compatible with multiple inks, which is used later for the production of arrays of multi-material lipid-based diffraction gratings. The resulting arrays can then be characterized by fluorescence microscopy and atomic force microscopy prior to immersion in water. Fluid phospholipid droplet arrays such as these can often be destroyed upon immersion in water under ambient conditions. However, they can be reliably immersed in a water-free nitrogen atmosphere [52]. We explain this by the idea that dehydrated DOPC is effectively frozen, and immersion in a water-free atmosphere prevents it from washing away. The immersion of gel-phase lipids, such as DPPC, does not require dehydration [58].

**Figure 4:**
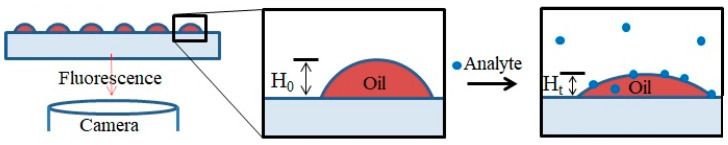
Schematic illustration of a lipid multilayer fluorescence assay. Fluorescently-labeled arrays of lipid droplets on a surface can be observed with a fluorescence microscope. Upon exposure to an analyte, the change in intensity or structure of the lipid arrays is monitored.

The experimental setup is depicted in Figure 4. Fluorescent lipid multilayers were patterned onto the surface. Upon exposure to the analyte in solution, the fluorescent intensity of the lipid multilayers changes. As a proof of concept, DOPC lipid multilayers were exposed to both 1 µM solutions of ethanol and the odorant lilial (Supplementary Figure S2). While the ethanol might have produced some mild disruption in lipid multilayer uniformity, lilial exposure elicited pronounced multilayer spreading, as evidenced by the lipid intensity increase between lipid multilayer dot structures and the decrease in lipid multilayer dot fluorescence intensity.

**Figure 5:**
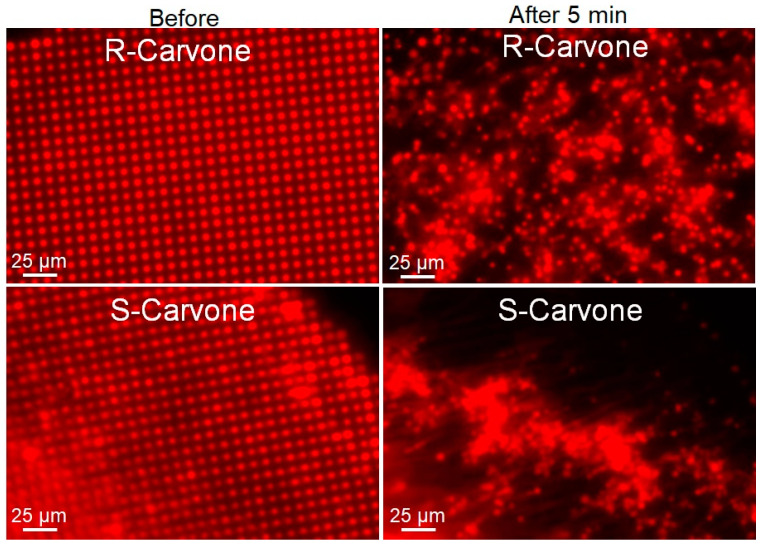
Lipid multilayer fluorescence assay upon exposure to R-carvone and S-carvone. Both enantiomers were found to significantly disrupt the lipid multilayer structures. Lipid dots exposed to 1 mM R-carvone demonstrated spreading and dissolution, while lipid multilayers exposed to 1 mM S-carvone tended to demonstrate more tension and remodeling effects.

To further study this phenomenon, 1 mM solutions of R- and S-carvone in 0.5 mol% DMSO were introduced to the lipid multilayer dot structures. As opposed to the hydrophilic odorant lilial, both carvone enantiomers perpetuated dewetting of the phospholipid multilayers that were physisorbed to the glass substrate (Figure 5). To further study the enantiomer selectivity by DOPC membranes, a dose–response was calculated by measuring the fluorescence intensity of multilayer dots both before carvone addition and after five minutes of odorant exposure (Supplementary Figure S3). ROIs of 25 dots from each sample were used to compute the average fluorescence intensity of a sample. We can see in the dose–response curve a difference in fluorescence intensity for concentrations of 10^−3^ M and lower, indicating less pattern disruption for R-carvone at lower concentrations than for S-carvone. This unique concentration dependence is consistent with what was observed in the FRAP data (Figure 2).

**Figure 6:**
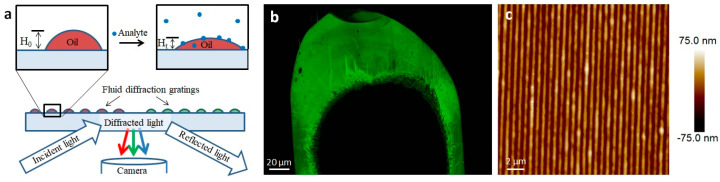
Lipid multilayer gratings. (**a**) Schematic showing the assay of exposing lipid multilayer gratings to an analyte while monitoring the diffraction from the arrays. (**b**) An example of an optical image of a DOPC-based lipid multilayer grating taken according to the setup shown in (**a**). (**c**) AFM image of a DOPC lipid multilayer grating.

**Figure 7:**
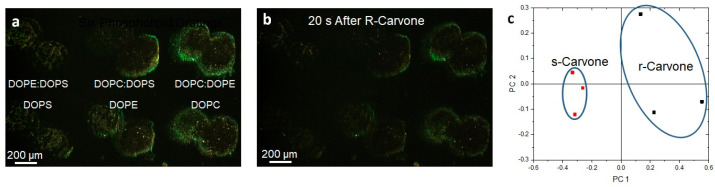
Lipid multilayer gratings respond to carvone vapors with enantiomer dependence. (**a**) Lipid multilayer gratings of DOPC, DOPE, DOPS, 1:1 DOPC:DOPE, 1:1 DOPC:DOPS, and 1:1 DOPE:DOPS were fabricated on a polystyrene surface. The sample was illuminated by white light from an angle and an image was taken of the light diffracted from the gratings as illustrated in Figure 5. (**b**) The gratings were then exposed to carvone vapors at saturation. (**c**) Utilizing three different experiments for each enantiomer, clustering was observed using multivariable analysis using 20 to 40 s time point data to construct the response of the gratings in PCA space.

While lipid multilayer dots suggested the odorants perpetuate dewetting of the lipid membranes from the surface in solution, it led to the question of whether the membranes were capable of discriminating enantiomers of a specific odorant through vapor. To answer this question, we used lipid multilayer sensor technology developed previously [54] to test the theory (Figure 6 and Figure 7). Upon analyte exposure to oil droplet gratings on the surface, analytes interact with the fluid oil gratings and dynamically change the heights. This height can be detected by label-free light diffraction and color camera capture. By printing lipid gratings in a polystyrene petri dish chamber, different carvone enantiomers could be administered to grating arrays and, using PCA, processed to determine if the entirety of the grating arrays responded differently to carvone enantiomer vapors.

Photomicrographs of the lipid gratings formed from six different formulations (DOPC, DOPE, DOPS, 1:1 DOPC:DOPE, 1:1 DOPC:DOPS, 1:1 DOPE:DOPS) of phospholipids printed onto the surface and exposed to carvone enantiomer vapor (three experiments for each enantiomer) are shown in Figure 7. The three mixed formulations are 1:1 by mol. Surprisingly, the enantiomers were able to be distinguished in principal component space. We previously demonstrated that varying the phospholipid compositions, when printed onto the surface and exposed to different volatile organic compounds and humidity, resulted in unique responses. The odorants S- and R-carvone were chosen because they bind different preferred odorant receptors. DOPC, DOPE, and DOPS were the compositions of phospholipids used to fabricate nanogratings because they are prevalent in the olfactory epithelium. The odor vapors were exposed to the phospholipids patterned as diffraction small gratings so that changes in optical diffraction could be monitored. Choosing time points at 20 s and 40 s after odorant application enabled the measurement of lipid multilayer responses to odorants and allowed us to generate a point in PCA space. Odor–lipid interaction profiles (like those generated in Supplementary Figure S4) demonstrated a change in lipid structure on the surface, and the response relationships between different odorant classes demonstrated the extent of lipid discrimination. An increase in response implies the lipid multilayers are swelling, or dewetting to increase in height, whereas a decrease in light intensity generally implies a spreading mechanism [52]. The data were analyzed using multivariable analysis to demonstrate the capability to distinguish the application of different odorants in PCA space. While PCA on carvone enantiomer response suggests an ability to distinguish enantiomers, we are unable to rule out possible artifact differences unrelated to chirality. More enantiomers should be used to further elucidate the relationship between how different classes of odorant enantiomers interact with lipid membranes. For example, lipid membrane enantiomer selectivity could depend on the functional group of the odorant and/or the phospholipid membrane composition.

## 4. Conclusions

The results presented here suggest that the lipid membrane may play a modulatory role in odorant discrimination. Given the kinetics of the lipid multilayer responses to the carvone enantiomers, lipid membranes may also serve a secondary function for odorant adaptation. FRAP and lipid multilayer assays demonstrated significant changes in bilayer properties upon exposure to odorants. In several cases, a marked difference between enantiomers was observed when comparing S-carvone and R-carvone, specifically at concentrations of 10^−3^ M and lower. One possible explanation could be based on the incorporation of the S-enantiomer into the lipid membrane based upon the chirality of both molecules. It is somewhat surprising that a difference between two enantiomers was reproducibly observed at lower concentrations but not higher concentrations. Dose–response effects in biological systems typically become stronger at higher concentrations [49]. The lipid-based enantioselectivity observed here may require further quantitative tests, possibly in different formats to eliminate the possibility of the synthetic enantiomer solutions being different in some other way beyond chirality. The observation that three different odorants have strong effects on the physical properties of model lipid membranes in the absence of protein suggests the possibility that the odor selectivity may be due to the membrane composition.

**Figure 8:**
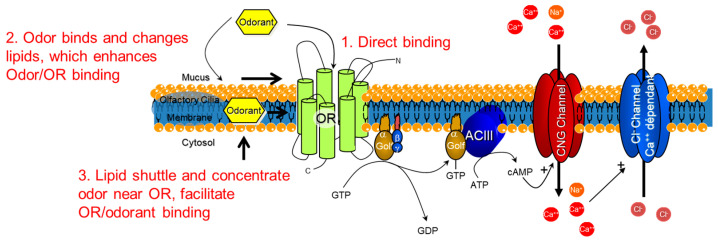
Possibilities for how the lipid bilayer membrane could assist in the molecular binding of odorants. Schematic depicting the odorant receptor (OR) G-protein-coupled receptor cascade and the three possibilities for molecular triggering of the cascade. 1. Direct binding of the odorant to the OR. 2. Lipid reorganization from odorants could provide access for the odorant to arrive in the binding site of the OR. 3. Lipids shuttle and concentrate odorants near the OR to facilitate OR/odorant binding. Golf = G-protein olfaction, ACIII = Adenyl cyclase 3, CNG = cyclic nucleotide.

The implications for odor recognition in vitro are that the supramolecular structure of lipid bilayer membranes in the olfactory epithelium may play a role in the signal detection and transduction process. Possible explanations for how an odorant could interact with the lipid membrane in order to help concentrate and enable the odor receptor to bind the odor are presented in Figure 8. An odor could directly bind (1) to the odor receptor through the mucus, or the odor could bind and change the fluidity or structure of the lipid membrane (2) to enhance odorant access to the odor receptors, depending on the partitioning of the odorant into the lipid bilayer. The accumulation of odorants in the lipid bilayer may lead to structural changes (3), for instance raft-like structures that could regulate one or more of the membrane-bound proteins illustrated in Figure 7 [27,34].

The possibility that the partitioning of a ligand into a lipid bilayer may trigger a pathway such as GPCR activation is a new way of thinking about receptors and membranes. Like odorants, many drugs tend to be lipophilic, and lipophilic drugs tend to be more potent than more polar drugs [66]. Furthermore, the lipid composition of lipid-based particles such as exosomes, liposomes, lipid nanoparticles, and other drug delivery vehicles is important for determining delivery properties, although in many cases the mechanism behind this remains unclear [67,68,69,70,71]. If the lipid composition of a bilayer leads to molecular recognition in vivo then that may lead to a better understanding of targeting and signal transduction and lead to innovative drug targets. Although it is challenging to test this hypothesis in vivo, considerations of lipid metabolism as well as model systems appear to be promising [72,73,74]. Further investigations of lipid composition and structure–function relations in vitro and in vivo may lead to new insights and possibly lipid-based therapeutic targets.

## Data Availability

Data sets generated by this study can be made available by contacting the communicating author.

## Supplementary Materials

The following supporting information can be downloaded at: https://www.mdpi.com/article/10.3390/membranes13020151/s1, Figure S1: DOPC supported bilayer FRAP response to lilial; Figure S2: Lilial Interaction with Lipid Multilayer Dots; Figure S3: Dose–Response of Carvone Enantiomers to Lipid Multilayers; Figure S4: Label-Free Lipid Multilayer Diffraction Gratings of Various Compositions’ Response to S- and R-Carvone Enantiomers.
