# Supplementary material for: Odor Discrimination by Lipid Membranes
Source: Membranes (Basel). 2023 Jan 24;13(2):151. doi: 10.3390/membranes13020151 (PMC9962961; doi:10.3390/membranes13020151)
Supplement: Supplementary file 1 [file membranes-13-00151-s001.zip › membranes-2168647-supplementary.pdf]

Supporting information for

Odor Discrimination by Lipid Membranes

Troy W. Lowry, Aubrey E. Kusi-Appiah, Debra Ann Fadool, and Steven Lenhert

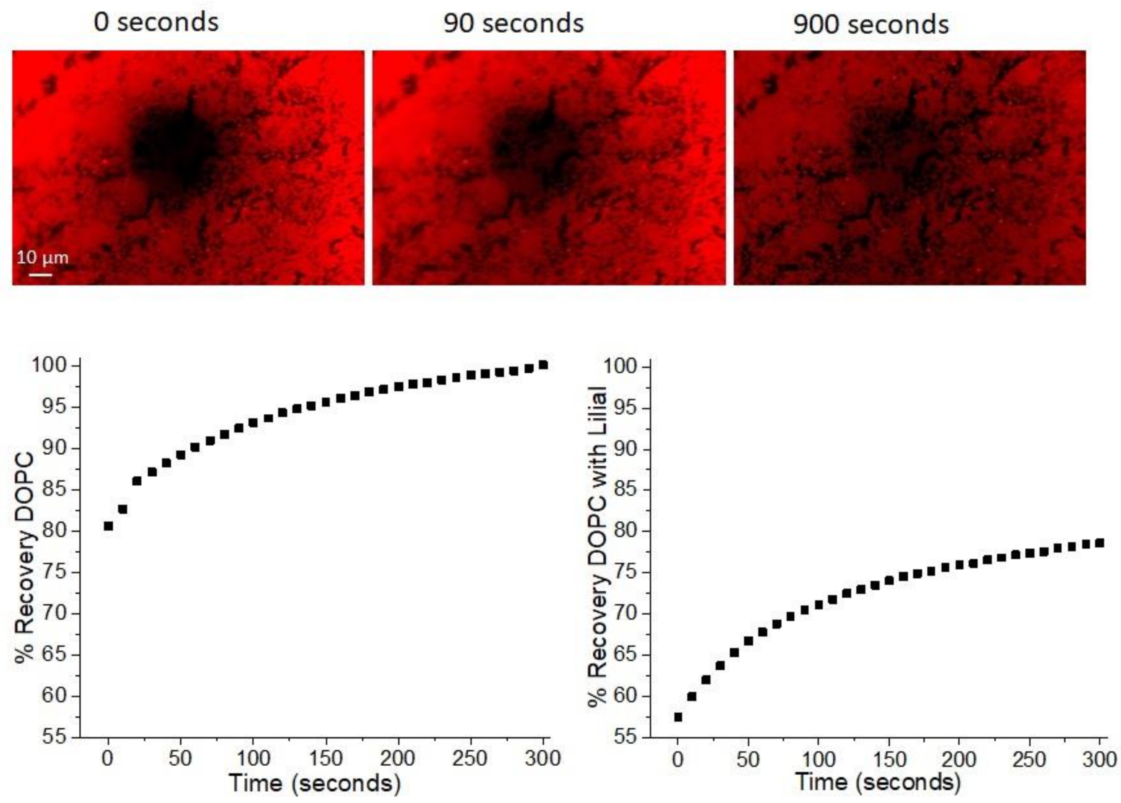

Figure S1. DOPC supported bilayer FRAP response to lilial. Fluorescent images show the overexposed area of a DOPC bilayer at application of lilial (0 seconds), after 90 seconds and after 900 seconds, indicating the odorant perpetuated lack of complete bilayer recovery. The graph on the left shows a control DOPC bilayer in water graph for fluorescent recovery. This graph reproduces the well-known DOPC recovery in literature. However in the prescence of lilial, DOPC bilayers have lower fluorescence intensity after bleaching and a lower recovery maximum intensity.

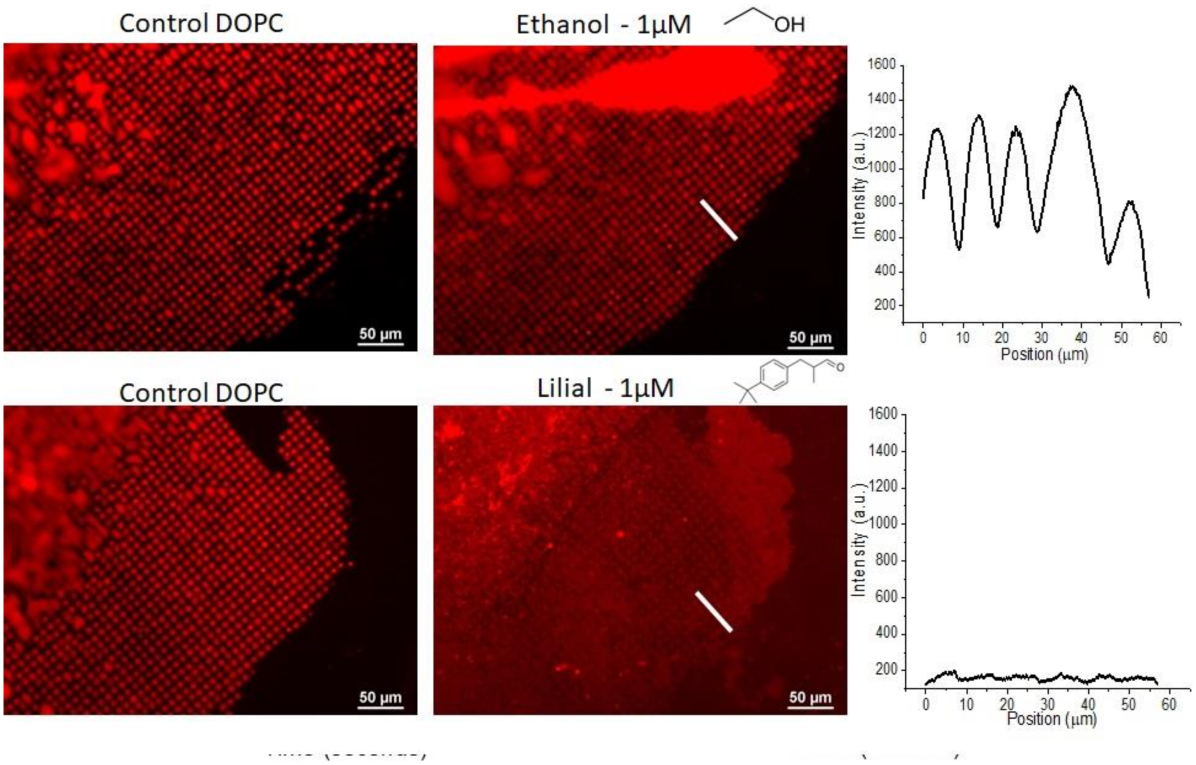

Figure S2. Lilial interaction with Lipid Multilayer Dots. Demonstrated here are fluorescent images of DOPC dot structures both before and after addition of 1µM ethanol and lilial, respectively. While ethanol application suggests multilayer swelling and minimal spreading, lilial produces a strong spreading effect that lowers the intensity of the dot patterns.

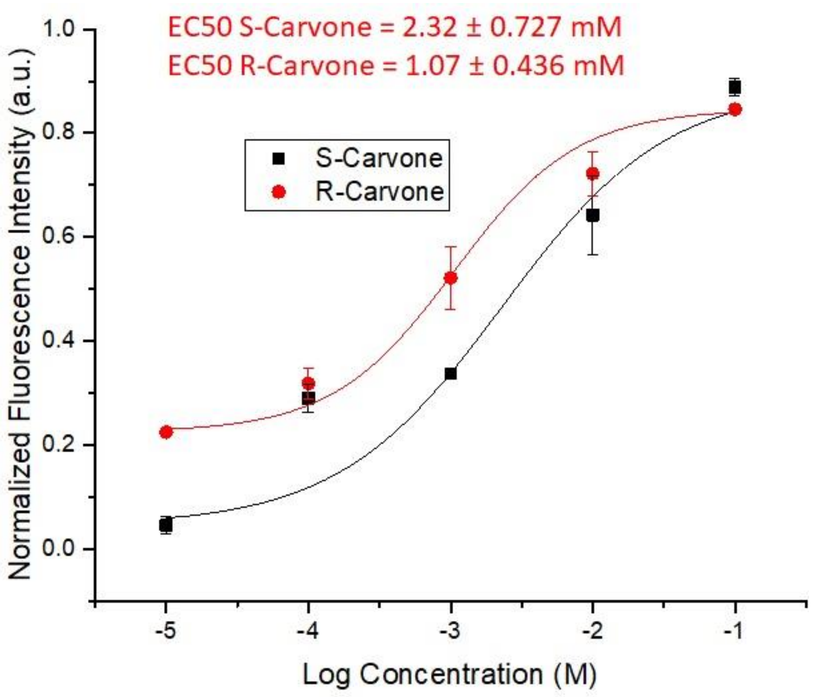

Figure S3. Dose Response of Carvone Enantiomers to Lipid Multilayers. DOPC multilayer dots were exposed to various concentrations of S- and R- enantiomers like in Figure 7. Average intensity of ROIs of 25 dots were drawn around the lipid multilayer patterns both before and after 5 minutes exposure to carvone enantiomer. Each concentration was repeated three times. Dose-response curve fitting distinguishes lipid multilayer response largely due to the stronger multilayer response in S-Carvone concentrations of 10^{-3}M and lower.

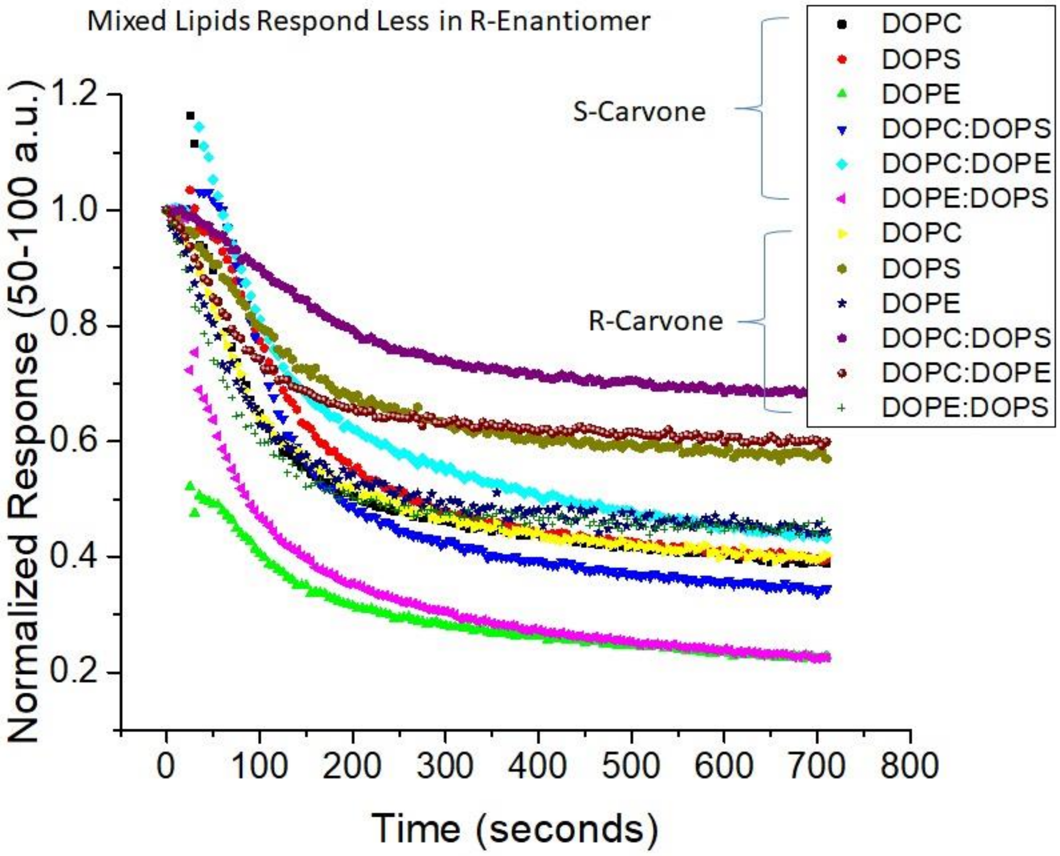

Figure S4. Label-Free Lipid Multilayer Diffraction Gratings of Various Compositions Response to S- and R-Carvone Enantiomers. The graph shows the lipid multilayer grating sensor response from the compositions listed in the legend of to 1mM S-Carvone and R-Carvone. The data shows mixed lipids responded less to the R-enantiomer than the S-enantiomer. The results support the possibility the chirality of lipid bilayers may play a role in selecting how different enantiomers interact in olfactory membranes.
